# VSUGAN unify voice style based on spectrogram and generated adversarial networks

**DOI:** 10.1038/s41598-021-03770-2

**Published:** 2021-12-21

**Authors:** Tongjie Ouyang, Zhijun Yang, Huilong Xie, Tianlin Hu, Qingmei Liu

**Affiliations:** 1 Modern Educational Technology and Practice Training Center, Innovation Laboratory for Undergraduate, Xiamen University, Xiamen, China; 2 School of Aerospace Engineering, Xiamen University, Xiamen, China; 3 College of Food and Biological Engineering, Jimei University, Xiamen, China

**Keywords:** Electrical and electronic engineering, Computer science

## Abstract

In course recording, the audio recorded in different pickups and environments can be clearly distinguished and cause style differences after splicing, which influences the quality of recorded courses. A common way to improve the above situation is to use voice style unification. In the present study, we propose a voice style unification model based on generated adversarial networks (VSUGAN) to transfer voice style from the spectrogram. The VSUGAN synthesizes the audio by combining the style information from the audio style template and the voice information from the processed audio. And it allows the audio style unification in different environments without retraining the network for new speakers. Meanwhile, the current VSUGAN is implemented and evaluated on THCHS-30 and VCTK-Corpus corpora. The source code of VSUGAN is available at https://github.com/oy-tj/VSUGAN. In one word, it is demonstrated that the VSUGAN can effectively improve the quality of the recorded audio and reduce the style differences in kinds of environments.

## Introduction

In recent years, popular online courses like SPOC and the applications of flipped classrooms have brought a large number of demands for regular course recording^1^. The audio records in different pickups and recording environments may generate additional background noise, which can be clearly distinguished by human ears after splice^2^, and lead to different sound qualities to affect recorded courses. Traditionally, this problem can be solved by manually adjusting the sound waveform or frequency spectrum during post-production or removing noise by denoise algorithms^3,4^. However, the involved post-production in regular course recording applications is usually unprofessional, and manual adjustment is time-consuming. Traditional denoising algorithms, such as spectral subtractive^5^, subspace^6^, statistical-model based^7^ and Wiener algorithms^8^ only remove part of the background noise and cannot solve the problem completely. Meanwhile, neuronal networks used for speech enhancement, such as SEGAN^9,10^, mostly focus on obtaining clear speech but fail to unify audio styles according to different environments.

Voice style unification also known as voice style transfer refers to combining a speaker's timbre, paralanguage (mood and intonation), and other characteristics into synthesized audio. Through decades of development, voice style transfer technology has obtained many achievements due to the applications of voice conversion technology. For instance, Valbret et al. proposed a method based on Pitch Synchronous Overlap and Add technology to realize voice transformation^11^, and Desai et al. used BP neural network to achieve speech conversion^12^. Thanks to the development of deep learning, especially long short-term memory networks, the performance has been improved significantly^13^. Moreover, in order to further enhance the quality of voice conversion, Donahue et al. presented wave GAN based on deep convolutional generative adversarial networks (DCGAN)^14^. However, existing generative adversarial networks (GAN) only solve the fixed one-to-one or many-to-many voice conversion scenarios. Once involving new speakers, the GAN should be retrained with transfer learning, which is reduplicative and unnecessary.

In the current study, a voice style unification model based on generated adversarial networks (VSUGAN) is established to unify voice style in different environments without retraining the network for new speakers. VSUGAN combines the style information from the audio style template and the voice information from the processed audio. In this method, background noise is also considered as a part of the audio style. The input consists of audio style template and noise-mixed audio, while the output is target-style audio. The contributions of this paper are as follows:

1. An audio style template is added as input. VSUGAN will adjust the audio style according to the template without retraining the network for new speakers.
2. By making reasonable assumptions about the training data, unsupervised learning is transformed into supervised learning, so that a large number of existing corpuses can be used in training VSUGAN.

### Spectrogram and voiceprint

The spectrogram is obtained by short-time Fourier transform (STFT) of the voice signal. The voice signal, i.e., waveform signal, is first divided into a number of overlapping frames according to the time window and then converted to the frequency spectrum by fast Fourier transform (FFT) frame by frame. Next, the frequency spectrum is arranged in the frame order to form a spectrogram^15^. The x-axis of the spectrogram denotes time, and the y-axis stands for frequency. The amplitude of a particular frequency at a specific time is represented by colors, where dark colors correspond to low amplitudes and brighter colors relate to progressively stronger amplitudes. The change of background noise and voiceprint after a piece of audio through VSUGAN can be clearly observed in the spectrogram. In the experiment, the librosa library was utilized to transfer the waveform signals to spectrogram with the FFT window size of 512.

**Figure 1:**
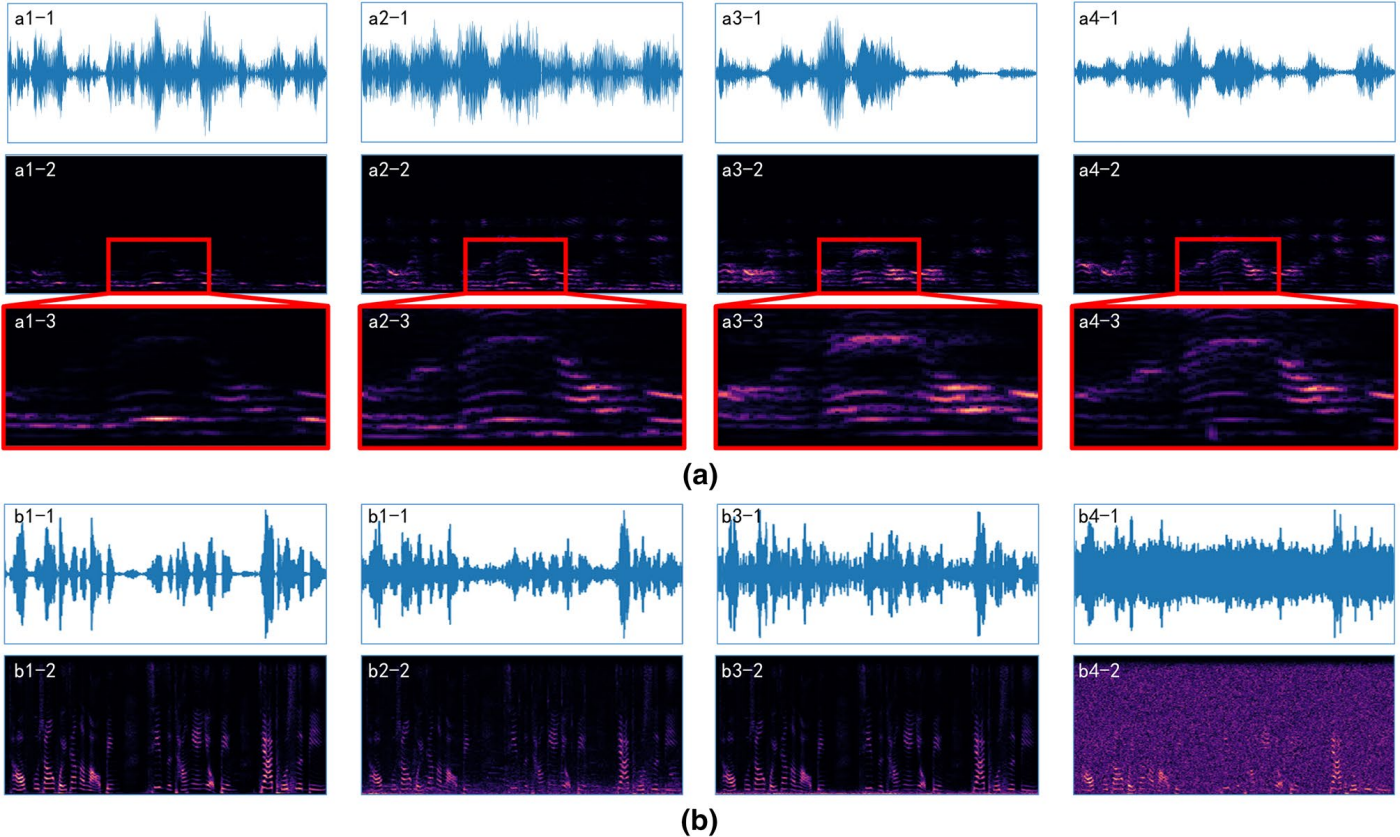
Effects of different recording environments and noises on audio. (**a**) Waveform (top row), spectrogram (middle row), and partial enlargement (bottom row) of recorded audio in different environments (each column means each environment). (**b**) Waveform (top row) and spectrogram (bottom row) of audio mixed with different noises (different columns).

Meanwhile, voice waveforms and spectrograms can reflect different voice recording effects in diverse recording environments. Figure 1a shows the waveform and spectrogram of a piece of audio recorded in four different environments. The corresponding recording settings are shown in Table 1. It can be seen from Fig. 1a1-1–a4-1) that the waveforms of the same sentence are disparate in four different environments. And the same sentence voiceprint are vary in detail from office or classroom environments (Fig. 1a1-2–a4-2, a1-3–a4-3).

**Table 1:** Different recording environments.

Group	Recording venue	Input devices
a1	Office	Sanako SLH-07 (3.5 in. socket)
a2	Office	Panasonic WX-4800 & X66
a3	Classroom	Sanako SLH-07 (3.5 in. socket)
a4	Classroom	Panasonic WX-4800 & X66

### Background noise

Background noise in different environments generates noise points with kinds of distributions and shapes on the spectrogram^16^. Figure 1b shows the waveforms and spectrograms of the same audio after overlaying different noises, where b1 is the original clean audio, b2 is mixed with cafeteria noise, b3 is combined with driving car noise, and b4 is integrated with Gauss white noise. It is clear from b2-2, b3-2, and b4-2 that the frequency of cafeteria noise and car noise is concentrated in the low-frequency area, while the Gauss white noise is distributed in the whole frequency area.

## Voice style unification based on GAN

### Algorithm and system structure

GAN consists of two parts: generator $G$ and discriminator $D$. Generator $G$ learns to map samples $z$ from some prior distribution ${P}_{z}$ to the target distribution ${P}_{G}$, However, ${P}_{G}$ is unknown, and discriminator $D$ is designed to judge the similarity between ${P}_{G}$ and prior distribution ${P}_{data}$. Discriminator $D$ is trained to distinguish samples $x$ from the prior distribution ${P}_{data}$ and the fake samples generated by $G$. Similarly, Generator $G$ is trained to make its output data $G(z)$ deceive the discriminator $D$ as much as possible, so that the discriminator $D$ cannot distinguish whether the data comes from the prior distribution ${P}_{data}$ or ${P}_{G}$. Alternately training $D$ and $G$ can increase their completion abilities until the data generated by $G$ meets the requirements^17^. This kind of adversarial training in classical GAN can be described as:

1 $$\underset{G}{\mathit{min}}\,\underset{D}{\mathit{max}}V(G,D)={E}_{x\sim {P}_{data}}\left[\mathit{log}\,D\left(x\right)\right]+{E}_{z\sim {P}_{z}}\left[\mathit{log}(1-D\left(G(z)\right))\right]$$

Assuming an extra input ${x}_{s}$ need to be added into the classical GAN, the output $G(z)$ of the generator can also have some properties related to ${x}_{s}$. Thus, the adversarial training with the addition of ${x}_{s}$ can be described as:

2 $$\underset{G}{\mathit{min}}\,\underset{D}{\mathit{max}}V(G,D)={E}_{x\sim {P}_{data},{x}_{s}\sim {P}_{data}}\left[\mathit{log}D\left(x,{x}_{s}\right)\right]+{E}_{z\sim {P}_{z},{x}_{s}\sim {P}_{data}}\left[\mathit{log}(1-D\left(G(z,{x}_{s}),{x}_{s}\right))\right]$$

In order to train VSUGAN more conveniently and stably, $Pearson{{\varvec{\upchi}}}^{2}$ divergence^18^ is applied to replace KL divergence in classical GAN:

3 $$ \begin{aligned} \mathop {min}\limits_{D} V\left( D \right) & = E_{{x\sim P_{data} ,x_{s} \sim P_{data} }} \left[ {(D\left( {x,x_{s} } \right) - 1)^{2} } \right] + E_{{z\sim P_{z} ,x_{s} \sim P_{data} }} \left[ {\left( {D\left( {G\left( {z,x_{s} } \right),x_{s} } \right)} \right)^{2} } \right] \\ \mathop {min}\limits_{G} V\left( G \right) & = E_{{z\sim P_{z} ,x_{s} \sim P_{data} }} \left[ {\left( {D\left( {G\left( {z,x_{s} } \right),x_{s} } \right) - 1} \right)^{2} } \right] \\ \end{aligned} $$

**Figure 2:**
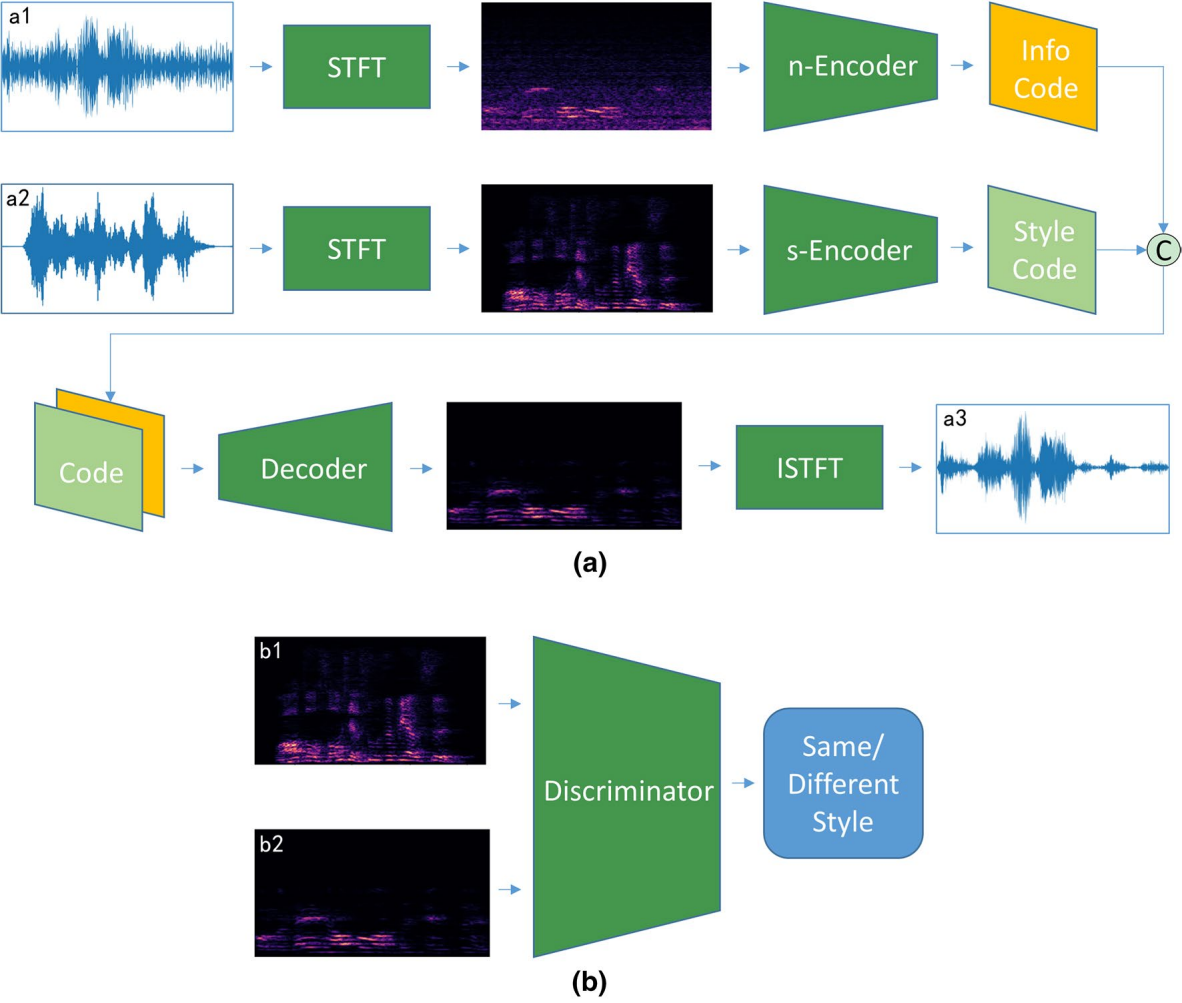
System workflows. (**a**) Generator workflow, where a1 is noise-mixed audio, a2 is style template audio, and a3 is target style audio. (**b**) Discriminator workflow, where b1 is the spectrogram of style template audio, and b2 is the spectrogram generated from the generator workflow.

The workflow of generator network $G$ is shown in Fig. 2a, which has two encoders and one decoder. The original utterances are down-sampled to 16,384 Hz and sliced into a group of segments with a length of 4 s. The segment length of 4 s is chosen because it is difficult to extract enough style information from shorter speech. The input of the encoder is the 257 × 513 × 1 spectrogram obtained by STFT of voice segments. One encoder (encoder for noise, termed as n-Encoder) is employed to extract the content information (Info Code) from the spectrogram of a-1 audio, and the other encoder (encoder for style template, using s-Encoder for short) is exploited to extract style information (Style Code) from the spectrogram of a-2 audio. Subsequently, content and style information, i.e., Info Code and Style Code, are combined and fed to the decoder. Next, the decoder outputs the spectrogram with a unified style, and the spectrogram generates the target audio through Inverse Short-Time Fourier Transform (ISTFT). Griffin Lim algorithm^19^ is used for generate phase signals in ISTFT.

At the same time, Fig. 2b illustrates the discriminator workflow. The input of the discriminator is the combination of the generator output spectrogram and the style template spectrogram. The output is the judgment of whether their styles are consistent.

### Generator configuration

**Figure 3:**
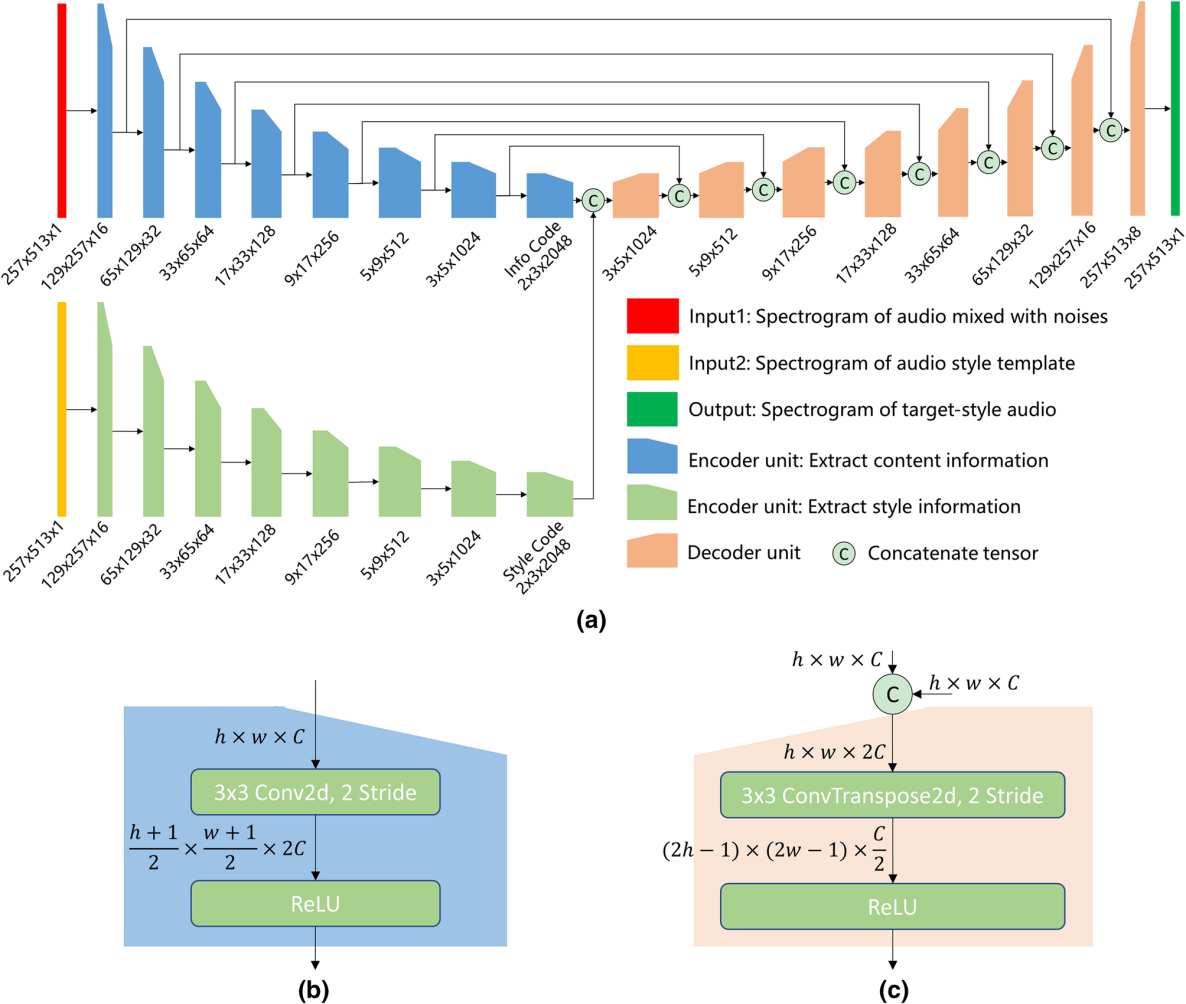
Generator configuration. (**a**) Generator structure. (**b**) Encoder unit. (**c**) Decoder unit.

The configuration of the generator is illustrated in Fig. 3. The n-Encoder and the s-Encoder which share the same structure in the generator network are designed to extract content and style information. They compress the input spectrogram of $257\times 513\times 1$ into the encoding information of $2\times 3\times 2048$ by eight encoder units, which are applied to downsample the image by a $3\times 3$ convolution kernel with a stride of 2, and no pooling layer is utilized similar to DCGAN^20^. After convolution, the data size changes from $(h,w)$ to $(\frac{1}{2}(h+1),\frac{1}{2}(w+1))$, and the convoluted data is activated by REctified Linear Unit (ReLU)^21^. Similar to the structure of the encoder, the decoder decodes the $2\times 3\times 4096$ encoded information into $257\times 513\times 1$ spectrogram through eight decoder units, which also apply a $3\times 3$ convolution kernel with a stride of 2 to upsample the image through fractionally strided convolutions. At the same time, skip connections are concatenating with the output result of the previous decoder and the input of the encoder^9^. Thus, skip connections are exploited to reduce the loss information and solved gradient explosion and gradient disappearance in training^22^.

### Discriminator configuration

**Figure 4:**
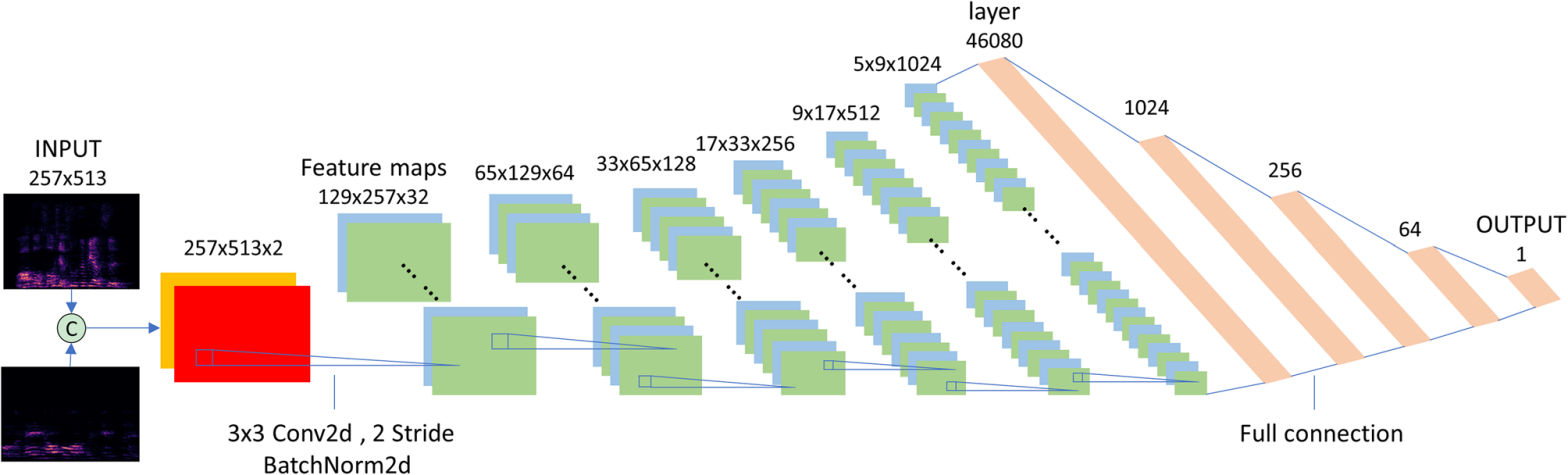
Discriminator configuration.

Figure 4 shows configuration of the discriminator. The discriminator concatenates two $257\times 513$ spectrograms into a $257\times 513\times 2$ tensor and generates $5\times 9\times 1024$ feature maps through six convolution layers by a $3\times 3$ convolution kernel with a stride of 2. The convoluted results are normalized by batch normalization and activated by ReLU. Then, the convoluted feature map is fed to the 5-layer fully connected network, and finally a score between [0, 1] is calculated to judge the styles similarity of two input voice segments.

### VSUGAN training

#### Data preparation

The data set used in VSUGAN is constructed by THCHS-30 corpus, which is an open-source Chinese speech corpus and contains 13,388 Mandarin sentences of 60 speakers. All the voice was recorded in a quiet office environment. In addition, the corpus includes three kinds of 0 dB noise: cafeteria noise (cafe), car noise (car), and Gaussian white noise (white)^23^. Firstly, the whole corpus is read and resampled to 16,384 Hz. And then, the speech and noise are segmented into segments per 4 s. According to different speakers, all speech segments are divided into the training set and testing set. The training set contains all the speech fragments of 51 randomly selected speakers, and the testing set includes the speech pieces of the rest nine speakers.

Let the set of all speakers as $A$, and the speech fragments set of one speaker $a$ as ${C}_{a}$. During the training, the spectrogram of the sample ${c}_{a}$ from ${C}_{a}$ is processed via image morphology algorithm to obtain $\stackrel{\sim }{{c}_{a}}$ by changing the voiceprint. Here, $\stackrel{\sim }{{c}_{a}}$ is used to destroy the original voice details and simulate the voiceprint changes caused by different pickups or environments. Meanwhile, the image morphology algorithm applied to process the spectrogram of the sample ${c}_{a}$ is randomly selected from the eight algorithms in Table 2.

**Table 2:** Image morphology algorithm and parameters.

SN	Algorithm	Filter window
1	Average filter	$3\times 3$
2	Median filter	$3\times 3$
3	Gaussian filter	$3\times 3$
4	Bilateral filter	Domain diameter = 3
5	Erode	$3\times 3$ Identity matrix
6	Dilate	$3\times 3$ Identity matrix
7	Open operation	$3\times 3$ Identity matrix
8	Closed operation	$3\times 3$ Identity matrix

Besides, a sample noise $n$ from noise set $N$ are mixed proportionally with $\stackrel{\sim }{{c}_{a}}$ to generate ${z}_{a}$ with the proportion $r=random(\mathrm{0,0.3})$:

4 $${z}_{a}=\stackrel{\sim }{{c}_{a}}+(r\times n)$$

where the set ${Z}_{a}$ including all ${z}_{a}$ is the mixed noise audio set of the speaker $a$.

In addition to ${c}_{a}$, another sample ${x}_{s}$ from ${C}_{a}$ is considered as a style template. Since the recorded environment and pickup of ${c}_{a}$ and ${x}_{s}$ are the same with speaker $a$, it can conclude the assumption that the styles of ${c}_{a}$ and ${x}_{s}$ are the same. Therefore, ${c}_{a}$ is used as a label to evaluate whether the generator output audio style is similar to ${x}_{s}$ in VSUGAN. It can transform the “judgment of style consistency” from an unsupervised learning problem to a supervised learning problemmake. The present design is very important for the discriminators' training.

#### Loss function

The loss function consists of two parts: one is the L1 loss judge the loss degree of information by calculating the output $G({z}_{a},{x}_{s})$ of the generator and ${c}_{a}$, the other is the discriminator loss to judge the loss degree of style.

##### L1 loss

Given the training data of the mixed noise audio ${z}_{a}$, the clean audio ${c}_{a}$, and the style template audio ${x}_{s}$, the L1 loss is defined as:

5 $${L}_{GL1}=\frac{1}{n}\sum_{i=1}^{n}|{\left({c}_{a}\right)}_{i}-{\left(G({z}_{a},{x}_{s})\right)}_{i}|$$

where $n$ is the number of elements in the matrix of the input spectrogram ${c}_{a}$ or the output spectrogram $G({z}_{a},{x}_{s})$ (the sizes of the two matrices are equal).

##### Discriminator loss

Given the training data of the mixed noise audio ${z}_{a}$, the clean audio ${c}_{a}$, and the style template audio ${x}_{s}$, the discriminator loss is denoted as:

6 $${L}_{D}={(D\left({c}_{a},{x}_{s}\right)-1)}^{2}+{(D(G\left({z}_{a},{x}_{s}\right),{x}_{s}))}^{2}$$

When training the generator, the output of discriminator $D$ is considered as a part of the generator loss to measure the loss of style:

7 $${L}_{GD}={(D\left(G\left({z}_{a},{x}_{s}\right),{x}_{s}\right)-1)}^{2}$$

##### Total loss

Combining the L1 loss and the discriminator loss mentioned above, the total loss of the generator is:

8 $${L}_{G}={L}_{GD}+(K\times {L}_{GL1})$$

where $K$ is termed as the hyper-parameter to control the weights of the two losses. Initially, $K$ was set to 100. But in VSUGAN, it can be observed that the ${\mathrm{L}}_{\mathrm{GD}}$ was one order of magnitude lower than the ${\mathrm{L}}_{\mathrm{GL}1}$. While *K* is set to 10, the two parts of the total loss achieve the best balance.

#### Training

Adam optimizer is used in both generator's training and discriminator's training in VSUGAN. The learning rate of generator is set to 0.001, and the value of discriminator is set to 0.0001. As a default, beta1 and beta2 are set to 0.9 and 0.999, respectively. VSUGAN is trained for 44 epochs using a batch size of 5. If the training is continued more than 44 epochs, the model will result in overfitting.

## Experimental results

### Evaluation metrics

Signal noise ratio (SNR) is an indicator of the amount of noise in the measured audio. The higher the SNR represent the lower amount of noise. SNR calculates the ratio of the output signal power $signal$ to the output noise power $noise$. The expression of SNR is formulated as:

9 $$SNR\left(dB\right)=10\,{\mathit{log}}_{10}\frac{{||signal||}_{2}}{{||noise||}_{2}}$$

For a group of training or testing data, the mixed noise audio ${z}_{a}$, clean audio ${c}_{a}$, style template audio ${x}_{s}$, and the generator output $G({z}_{a},{x}_{s})$ are used as signal. The difference between the audio to be measured and ${c}_{a}$ is used as noise, the formulation changes to:

10 $$ \begin{aligned} SNR_{out} & = 10\,log_{10} \frac{{\left| {\left| {c_{a} } \right|} \right|_{2} }}{{\left| {\left| {out - c_{a} } \right|} \right|_{2} }} \\ SNR_{{z_{a} }} & = 10\,log_{10} \frac{{\left| {\left| {c_{a} } \right|} \right|_{2} }}{{\left| {\left| {z_{a} - c_{a} } \right|} \right|_{2} }} \\ \Delta SNR & = SNR_{out} - SNR_{{z_{a} }} \\ \end{aligned} $$

${SNR}_{out}$ is the SNR of the generator output, and ${SNR}_{{z}_{a}}$ is the SNR of ${z}_{a}$, $\Delta SNR$ is the different between ${SNR}_{out}$ and ${SNR}_{{z}_{a}}$. Style unification is more effective when $\Delta SNR$ is higher.

Mel Cepstral Distortion (MCD) is usually utilized in voice conversion tasks to calculate the similarity between target voice and converted voice. Using Mel-Cepstral coefficients (MCEP)^24^, MCD calculates the Euclidean distance^25^ between the Mel-cepstrum of two voice signals. While the MCD value is lower, the similarity between the two voices is higher. In the testing of VSUGAN, the original clean audio ${c}_{a}$ was used as a reference to calculate the MCD of mixed noise audio ${z}_{a}$ and generator output $G({z}_{a},{x}_{s})$. In the implementation, pyworld library, pysptk library, and librosa library were used to analyze MECP and MCD^26^. In the present work, MCD of the generator output is named ${MCD}_{out}$ and MCD of the ${z}_{a}$ is named ${MCD}_{{z}_{a}}$. $\Delta MCD$ equals ${MCD}_{{z}_{a}}$ minus ${MCD}_{out}$. The $\Delta MCD$ is positively correlated with model performance.

In order to further evaluate the performance of the VSUGAN, two additional indicators (PESQ^27^ and STOI^28^) were chosen. And Pypesq and Pystoi libraries are used for analysis of PESQ and STOI.

### Training Results

**Figure 5:**
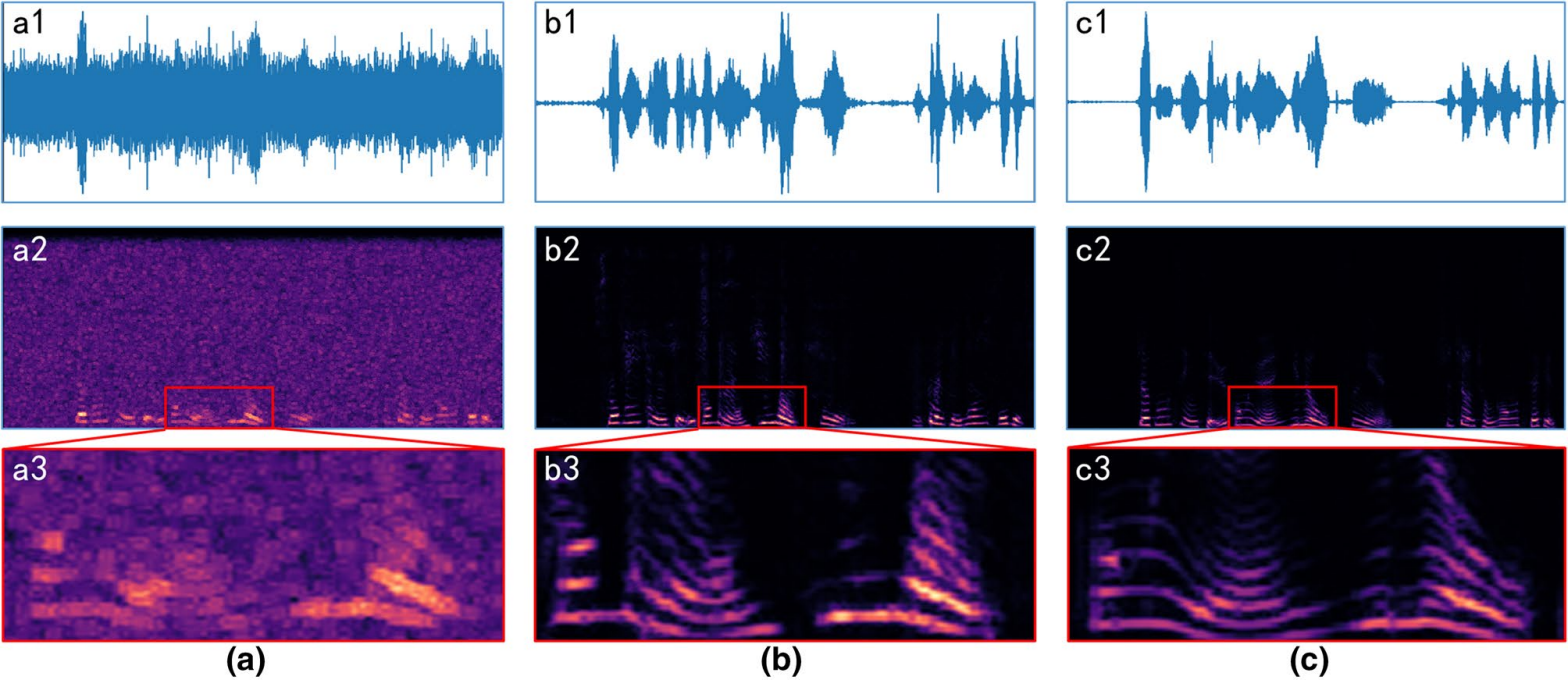
The processing effect of the speaker D13 voice mixed with 30% Gaussian white noise. (**a**) Noise mixed audio. (**b**) Output audio of network’. (**c**) Original audio. The top, middle, and bottom rows represent the waveform, spectrogram, and partial enlargement, respectively.

Figure 5 shows the effect of a piece of audio processed after the network. Figure 5a is the audio input of the network with the waveform (a1), spectrogram (a2), and partial enlargement (a3). The audio is a sample from the testing set with destroyed voiceprint details on the spectrogram, mixed with 30% Gaussian white noise. Figure 5b illustrates the audio output from the generator, where another clean audio of the same speaker is used as the style template to perform style consistency calculation on the input audio of Fig. 5a. And the original audio of Fig. 5a is shown in Fig. 5c. Through comparing (a2) and (b2) in Fig. 5, it can be seen that the background noise is basically filtered out by the VSUGAN. Besides, it is clear that the damaged voiceprint details in (a3) are recovered to a certain extent in (b3), by comparing (a3), (b3), and (c3) in Fig. 5.

**Figure 6:**
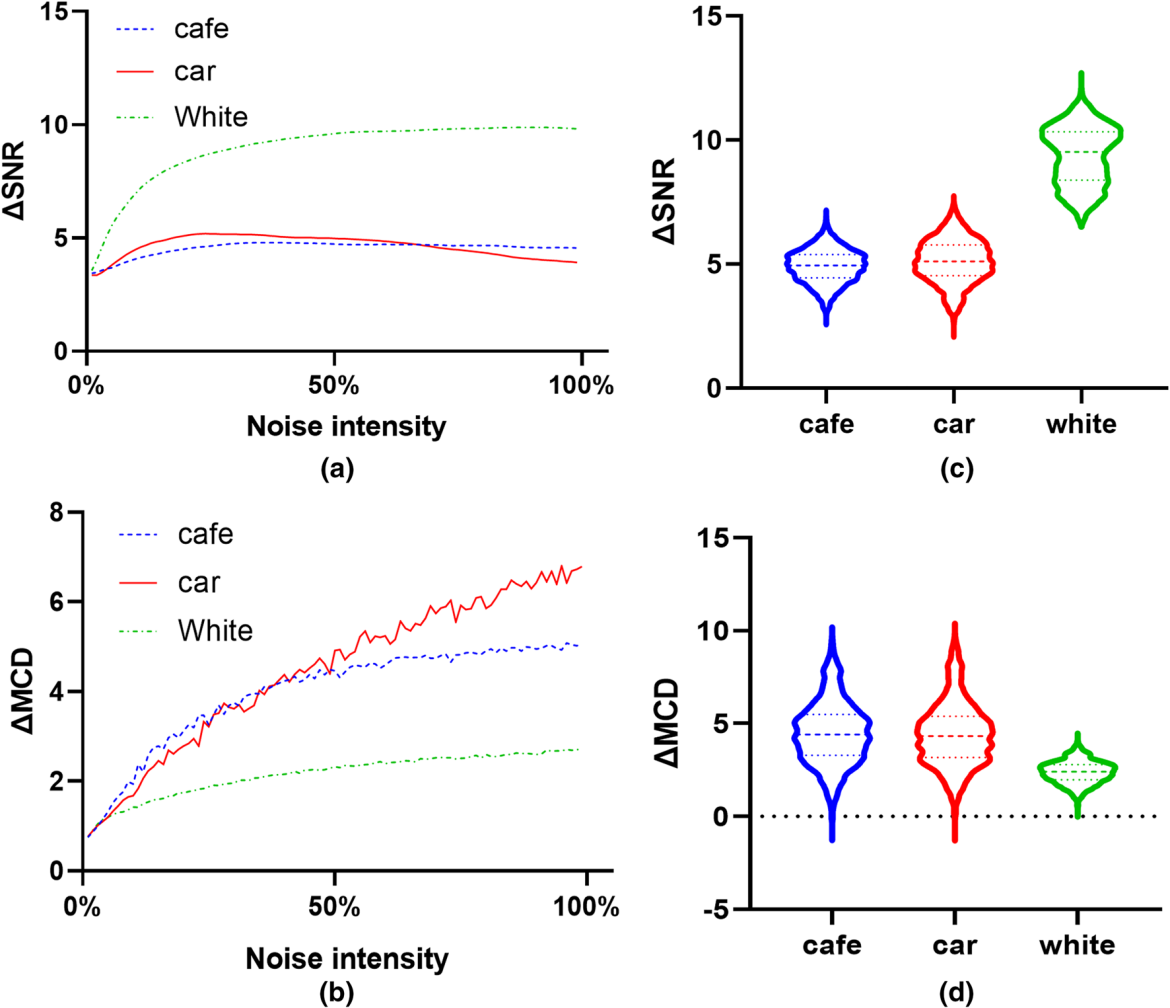
The ΔSNR and ΔMCD statistics of the testing set. (**a**) ΔSNR with 0–100% background noise intensity. (**b**) ΔMCD with 0–100% background noise intensity. (**c**) ΔSNR with 30% background noise intensity. (**d**) ΔMCD with 30% background noise intensity.

The statistics of ΔSNR and ΔMCD of testing set audio with different background noise is calculated in VSUGAN (Fig. 6). In the testing set, the input of (a) and (b) in Fig. 6 is nine sample audio from nine speakers whose original voiceprint details are destroyed by the image morphology algorithm on the spectrogram and then mixed with noise, i.e., cafe noise, car noise, and white Gaussian noise. The mixed noise intensity is 0–99% of 0 dB with increasing 1% every intensity. Therefore, $\Delta SNR$ and $\Delta MCD$ of nine input and output audio are obtained. With the larger $\Delta SNR$ and $\Delta MCD$, the output style is more consistent. From Fig. 6a, b, we can find that the values of $\Delta SNR$ and $\Delta MCD$ have significant correlation with noise intensity. When the noise intensity exceeds 30%, $\Delta SNR$ value is about 4–10 dB, where Gaussian white noise is significantly greater than other two kinds of noise. On the contrary, the $\Delta MCD$ with Gaussian white noise is lower while the noise intensity exceeds 30%. Due to the repaired voiceprint in VSUGAN, SNR and MCD of output audio are better than that of input audio, even if the mixed noise intensity is 0%.

In Figs. 6c, d, all original audio in the testing set which is mixed with three kinds of noise in the fixed intensity after destroying the voiceprint details are used as the network's input audio. As shown in Figs. 6c, $\Delta SNR$ of the audio mixed with Gaussian white noise is significantly higher than mixed with other two kinds of noise. However, $\Delta MCD$ value of the audio mixed with Gaussian white noise is lowest in three kinds if noise (Fig. 6d). Thus, it can be seen that VSUGAN has different degrees of improvement for types of noise.

Along with the testing set of THCHS-30, VCTK-Corpus^29^ and NoiseX-92 dataset were applied to validate the performance of VSUGAN. NoiseX-92 which have 15 kinds of noise (including white noise, pink noise, and vehicle interior noise) is part of the Signal Processing Information Base^30^. In the performance test, ten audio samples of nine speakers were randomly sampled from two datasets (90 audio samples per dataset). These audio samples were also mixed with the noise of random intensity after the voiceprint details were destroyed. As shown in Table 3, the noise in group1 and group2 has the same source as the noise mixed in during training. The noise in group3 was selected randomly from the NoiseX-92 dataset and has not been trained. Group4 has the same corpus as group1. In order to simulate the real acoustic environment, rir_generator^31^ is used in group4 to generate reverberation without the image morphology algorithm. This mixed-noise audio was fed into VSUGAN, and the average values of PESQ, STOI, SNR and MCD from output audio are calculated. The better audio quality is reflected in higher value of PESQ/STOI/SNR and lower value of MCD. It is worth mentioning that only THCHS-30 is utilized during VSUGAN training. SEGAN is used as a baseline for comparison, and the training of SEGAN use the same data set and method from Pascual et al^9^.

**Table 3:** Audio processing effect statistics in different corpora.

		SNR	MCD	PESQ	STOI
Group1 THCHS30	Noisy	− 4.24	6.88	1.95	0.76
	SEGAN	− 0.6	4.77	1.4	0.54
	VSUGAN	− 0.03	4.19	2.13	0.73
Group2 VCTK	Noisy	− 3.51	7.07	1.98	0.82
	SEGAN	− 0.79	6.04	1.45	0.61
	VSUGAN	− 0.03	5.36	2.06	0.8
Group3 VCTK_Noise92	Noisy	− 3.01	8.03	2.09	0.83
	SEGAN	− 1.05	6.75	1.65	0.65
	VSUGAN	− 0.01	5.9	2.03	0.8
Group4 THCHS30_Reverb	Noisy	− 1.48	4.84	3.14	0.08
	SEGAN	− 0.61	5.05	2.05	0.06
	VSUGAN	− 0.06	4.57	2.3	0.11

The experimental results is shown in Table 3. Compared with input noisy, the indicators of SNR and MCD have significantly improved in all four groups. And PESQ value has advanced in group1 and group2 but not in group3 and group4. The STOI value has few difference between group1–group3, while is increased slightly in group4. Thus, the current VSUGAN has stable performance in different data sets.

For the reproducible research, our source code was uploaded to Github (https://github.com/oy-tj/VSUGAN). The data set and trained model are shared on an online disk (https://pan.baidu.com/s/1RwvpwZjSET7hrLvpfvNizA password: 4y8a).

## Conclusion

In the present study, we proposed and implemented a model based on GAN by combining the style information extracted from the style template audio and the voice information extracted from the audio. VSUGAN without training for extra speakers generates the audio as same style as the template. VSUGAN is trained with THCHS-30 corpus and tested on two open-source corpora. The experimental results demonstrated that VSUGAN can effectively improve the quality of the recorded audio and reduce the style differences in kinds of environments.
